# Astrocyte Dysfunctions and HIV-1 Neurotoxicity

**DOI:** 10.4172/2155-6113.1000255

**Published:** 2013-11-19

**Authors:** Hoai Ton, Huangui Xiong

**Affiliations:** Neurophysiology Laboratory, Department of Pharmacology and Experimental Neuroscience, University of Nebraska Medical Center, Omaha, NE 68198-5880, USA

**Keywords:** Astrocytes, HIV-1 associated neurotoxicity, K^+^ channels, Glutamate uptake, Blood brain barrier, Gap junctions

## Abstract

Astrocytes play an important role in maintaining an optically suited milieu for neuronal functionality, and are involved in the progression and outcome of many neuropathological conditions. It becomes increasingly evident that astrocytes are significant contributors to HIV-1 associated neurological disorders by modulating the microenvironment in the central nervous system and releasing proinflammatory cytokines. Recent studies have revealed direct metabolic interactions between neurons and astrocytes observed particularly in HIV-1-associated neurological disorders by which astrocytic dysfunctions disregulate extracellular K+ homeostasis, intracellular calcium concentration, glutamate clearance, and blood brain barrier integrity and permeability. Such dysfunctions are amplified via gap junctions, directly or indirectly impacting surrounding neurons and significantly contributing to the pathogenesis of HIV-1-associated neuropathology. In this review, we tentatively address recent progresses on the roles astrocytes may play in HIV-1-associated neurotoxicity.

## Introduction

The human immunodeficiency virus type one (HIV-1) invades the central nervous system (CNS) at early stage after primary infection [1], which may result ultimately in the development of several types of neurological disorders. Such HIV-1-associated neurological disorders affect about 50% of infected individuals [2]. Characteristics of neurological disorders encompass cognitive disturbances, behavioral changes, and motor impairment [3], which are termed collectively as HIV-1-associated neurocognitive disorders (HAND) [4]. Despite the advent of combined antiretroviral therapy (cART), the prevalence of HAND continues to rise due to sustained HIV-1-infection of mononuclear phagocytes (MP, brain macrophages and microglia), the emergence of resistant viral phenotypes, poor drug penetration of blood-brain barrier and increase in patient lifespan. This indicates that HAND remains highly prevalent and continues to be a significant public health problem [5–7]. As cART cannot provide complete protection from HIV-1-induced neuronal injury and there is no efficacious treatment for HAND at present, mainly because the precise mechanisms underlying HIV-induced neuronal injury are not well understood. Thus, investigating molecular and cellular mechanisms of HIV-1-induced neuronal injury may provide valuable insights into the pathogenesis of HAND, and is essential for the development of preventive and therapeutic strategies.

The cellular basis and mechanisms underlying HAND is complex and have so far remained elusive. It has long been believed that neuropathogenesis of HAND revolves solely around metabolic processes induced by virus-infected and activated MPs. This is underscored by the findings that the abundance of macrophages in the brain appears to better correlate with HAND than the extent of brain infection [8,9], implicating that an increase in trafficking of macrophages to the brain may be associated with the development of HAND. In addition to pathogenic role of MPs, it is becoming increasing evident that astrocytes are also important players in the pathogenesis of HAND and other neurological disorders.

Astrocytes are the most abundant cell type within the CNS and play an important role in CNS homeostasis and function. They are also the target cells for immune mediators, bacterial toxins and viruses (such as HIV-1) that induce reactive astrogliosis, a common feature seen in many neurological disorders including HAND [10–12]. Astrocytes respond to pathological challenges by rapid activation, not only at the site of challenge but also in the surrounding neuropil [13]. Studies have shown an up-regulated expression of many proteins in activated astrocytes. These include cytokines, growth factors, cell surface molecules and extracellular matrix proteins [11]. While many of these molecules function as neurotrophic agents under certain circumstance, ample evidence support an important role for astrocytes as mediators of HIV-1-induced neuronal injury [11,14,15]. However, the mechanisms for how activated astrocytes induced neuronal injury are not fully understood. In this article, we try to highlight astrocytic dysfunctions and their roles in HIV-1 neurotoxicity, which potentially contribute to the pathogenesis of HIV-1 associated neurocognitive impairment.

## Astrocytosis

Astrocytosis, activated astrocytes, is an abnormal increase in the number and size of astrocytes in response to the CNS injury and disease. It has been observed in brain trauma, infection, stroke and other neurodegenerative diseases. Astrocytosis is characterized by hypertrophy, cell proliferation, progressive alteration in molecular expression and up-regulation of glial fibrillary acidic protein (GFAP) expression [16]. Astrocytosis may be beneficial or harmful in proximity to neural and non-neural cells. They undergo a spectrum of changes that may alter astrocytic activities and affecting surrounding cells.

HIV-1 infection of astrocytes has been noted in pediatric, and to a lesser extent, adult HIV-1 associated encephalopathy. Such infection is restricted at both virus entry and post-entry viral gene expression [17]. It has been shown that in HIV-1 infected individuals astrocytosis can be triggered not only by virus infection [18], but also induced by viral proteins or other macrophage products [19]. HIV-1 Tat-mediated brain lesions have also been reported to be associated with an increase of astrocytosis. Thus, astrogliosis is considered a striking pathological feature in HIV-1 infected brain [20]. Increasing evidence indicates astrocyte dysfunction during chronic HIV-1 CNS infection and immune activation play an important role in HIV-associated neuropathogenesis.

Astrocytes are identified by GFAP expression, which is an intermediate filament protein in the main processes and the soma. GFAP is generally considered to be a specific marker for astrogliotic response [21]. This protein is supposed to be involved in cell communication, cell migration, mitosis and cytoskeletal changes [22]. Astrocyte activation is characterized by elevated GFAP expression and this up-regulation is caused by diverse neurological insults [23], it is considered to be a hallmark feature observed in neurodegenerative diseases such as Alzheimer’s disease, Parkinson’s disease and HIV-1 infection [24]. Increased GFAP expression has also often been assumed to result from astrocyte proliferation [25]. Notably, astrocytes in GFAP knockout animals have interupted neuronal plasticity [26]. Astrocytosis is linked to productive infection of CNS and correlated with the cognitive disorders. It has also been shown that intracellular expression of HIV-1 Tat protein in astrocyte is linked to astrocytosis characterized by up-regulation expression of GFAP, astrocyte dysfunction, and neuronal death [24]. In addition, there is evidence that HIV-1 infected reactive astrocytes, together with activated microglia, may play a part in neuronal damage through the release of several inflammatory factors [18].

Activated astrocytes release neurotoxic factors including excitatory amino acids (EAAs) (such as glutamate) and TNF-α [27]. It is reported that gp120 up-regulates nuclear factor erythroid derived 2-related factor 2 (Nrf2), a basic leucine zipper transcription factor, which is involved in regulating the antioxidant defensive mechanism [28]. In addition, it has been shown that gp120 induces expression of IL-6 [29], IL-8 [30] and CCL5 in astrocytes [31] through a nuclear-kappa β-dependent mechanism. These results suggest that neuroinflammation in HIV-1-infected individuals could be mediated via the NF-kβ pathway. However, the dysfunction of activated astrocyte and their potential contributions to HIV-1 excitotoxicity and consequent CNS disorders is an area that warrants further insight.

## Gap Junction and HIV-1 Infected Astrocytes

Gap junctions are the means of intercellular contact between two cells, which allows intercellular passage of ions and many molecules such as K^+^, second-messenger molecules, various metabolites and small peptides between the cytoplasm of adjacent cells. Astrocytes are interconnected to each other via gap junctions along with neurons and other glial cells via heterotypic gap junctions [32]. Gap junctions are composed of two aligned “hemi channels” or connexous, which form a specific pathway for the communication across the intercellular space and provide a specific route for propagating waves of ionic and metabolic signaling between cells [33]. On the other hand, despite the low numbers of HIV-infected astrocytes (8.2% *in vivo* and 4.7% in vitro using an SIV model) [34], significant changes in BBB integrity, Ca^2+^ concentration, glutamate clearance, or even cell death in un-infected astrocytes occur. It is proposed that HIV-infected astrocytes spread toxic signals to uninfected astrocytes by gap junction channels [35].

Gap junctions play a critical role in transmitting and thereby amplifying toxic signals originating from HIV-infected astrocytes to neighboring cells. It has been shown that Tat-induced BBB dysfunction is associated with altered expression of tight junction proteins zone occuldens (ZO-1), and junctional adhesion molecule-2 (JAM-2) [36]. It is also demonstrated that HIV infection of human astrocytes disrupts BBB integrity by a mechanism that is dependent on functional gap junctions [34,37]. Moreover, a low number of HIV-infected cells have been shown to affect profoundly BBB integrity, which is associated with dysregulating endothelial survival, stability of astrocytes end feet, and signaling [34]. Interestingly, Gap junction blockers abolished apoptosis in uninfected astrocytes [35].

It is noteworthy that gap junctions have critical contributions to astrocyte functionality. Astrocyte gap junctions have been shown to modulate ATP release and glutamate homeostasis [38,39]. In addition, gap junctions are also noted to create a buffering system that is responsible for the dissipation of focal increases of extracellular K^+^ resulting from neuronal activity [40]. Notably, BBB disruption induced by infected astrocytes was reduced by gap junction blockers, meaning that functional gap junction channels potentially mediate BBB integrity and permeability [34]. Therefore, mechanism of toxicity within the brain is generated by the low numbers of HIV-infected astrocytes and amplified to neighboring un-infected astrocytes by gap junctions, playing a significant role in HIV-1 CNS dysfunction [35].

## K^+^ Channels in Astrocytes

Astrocytes are multifunctional cells that are indispensable for neuronal survival and function, especially in the regulation of extracellular K^+^ [41]. During neuronal activity, K^+^ ions are transferred from the cytoplasm to the extracellular space and cause an increase in [K^+^]_0_ which, if uncorrected, would induce the depolarization of neuronal membranes and the interruption of axonal conduction and synaptic transmission [42]. The clearance of extracellular K^+^ by astrocytes takes place through a combination of active uptake, co-transport and through K^+^ channels. Many studies have characterized that astrocytes have more hyperpolarized resting potential than that of neurons due to their expression of a wide variety of K^+^ channels [43], which are located in astrocyte synapses and at end-food processes around capillaries [42] to mediate spatial K^+^ buffering and regulate neural activities [44].

It has been strongly implicated that inward rectified K^+^ channels (Kir) play roles to assist in spatial buffering of the extracellular K^+^ released during neuronal firing. Especially, Kir4.1 channels have been proven to be a specific glial Kir subunit [45], suggesting that they may have a specific function in glial K^+^ regulation [46]. It is reported that Kir4.1 is responsible for hyperpolarization of astrocytes and is likely to play a critical role in K^+^ buffering. Reduced astroglial Kir current was observed in many pathological conditions such as traumatic hippocampal glia [47], ischemic brain injury [48], cortical dysplasia [49] and in neurosurgical specimens from epilepsy patients [50]. In addition, Kir4.1 channels also appear to be responsible for the major resting K^+^ conductance. Immunohistochemistry suggested that Kir4.1 is localized preferably on astroglial processes, ensheathing synapses and blood vessels [51]. Notably, genetic down-regulation of Kir4.1 impairs the ability of astrocytes to remove K^+^ and glutamate in the extracellular space *in vitro* [52] and *in vivo* [53]. These results suggest a clear role for Kir4.1 in astrocyte K^+^ buffering and glutamate uptake.

In contrast, the function of outward K^+^ current in astrocyes is not very clear. It has been proposed that the outward K^+^ current in astrocytes also contribute to special buffering, especially delayed rectified K^+^ current [54]. However, It remains to be determined on which type(s) of K^+^ channels contribute(s) significantly to the capabilities of cortical astrocytes in K^+^ buffering and glutamate uptake and how these K^+^ channel functions are affected during HIV-1 brain infection-leading to HAND pathogenesis.

## Glutamate Clearance

Glutamate is the primarily excitatory neurotransmitter in the mammalian CNS [55]. Rapid removal of glutamate for the extracellular space is essential both for survival and normal functioning of neurons. Approximately 40% of all neuronal synapses in the brain are glutamatergic [56]. Thus, regulation of glutamate is crucial for optimal glutamatergic neurotransmission, which may otherwise cause to neuronal death by glutamate excitotoxicicty [55]. Uptake of glutamate is mainly mediated by transporters localized at astrocytic membranes. Although glutamate transporters are expressed in both astrocytes and neurons, astrocytes are the cells primarily responsible for glutamate uptake. It is estimated that astrocyte glutamate transporters are responsible for up to 90% of glutamate uptake in the brain [22].

Glutamate excitotoxicity has been proposed as one of potential mechanisms for neuronal injury in several neurological diseases including amyotropic lateral sclerosis, Alzheimer’s disease [42] and HAND [57]. Exposure of cultured astrocytes to HIV-1 and gp120 impaired transport of extracellular glutamate by astrocytes [58]. Glutamate uptake inhibition was also observed during infection of feline astrocytes with feline immunodeficiency virus (FIV) [59] and in human astrocytes co-cultured with HTLV-I-infected T cells [60]. These results are in line with the findings of HIV-1 inhibition of glutamate transporter expression such as EAAT2 in astrocytes [58]. In addition, HIV-1 envelope proteins gp120 and gp41 have also been shown to impair glutamate uptake by primary human astrocytes [61], suggesting that disruption of astrocyte glutamate transport may be a common cytopathogenic activity mediated by some neurotropic retroviruses.

Glutamate transport is tightly regulated through transporter expression in astrocytes during brain development and in mature brain, and the levels of expression may be dys-regulated in diseases [62]. Five glutamate transporters of the SLC gene family, termed excitatory amino acid transporters 1 to 5 (EAAT1-5), have so far been identified in human brain [55]. Of these transporters, EAAT1 and 2, or glutamate transporter 1 (GLT-1) and L-glutamate/L-aspartate transporter (GLAST) in rodents, are expressed predominantly in astrocytes in the CNS and are believed to mediate most glutamate uptake in the brain. Expression of transporters EAAT1 and 2 was reduced in response to HIV-1, gp120 [58], and Tat [24]. These findings were in an agreement with the experimental results showing Tat-expression in astrocytes markedly impaired their ability in glutamate uptake [24]. These results indicate that HIV-1 and viral proteins may down-regulate expression and function of transporters EAAT1 and 2, resulting in glutamate excitotoxicity and neurodegeneration associated with HAND.

The synthesis and release of glutamine by astrocytes in part of a biochemical shuttle mechanism termed as the glutamate-glutamine cycle. After the release from the presynaptic terminal, glutamate is taken up mainly by astrocytes, and then converted to glutamine inside the astrocyte by the ATP-consuming reaction catalyzed by glutamine synthetase, an enzyme which is present in astrocytes but not in neurons [38]. It has been shown that GLT1 deficiency resulted in synaptic glutamate accumulation and subsequent excitotoxicity in mice [63]. Moreover, the reduction of EAAT1 and 2 expression and function in reactive astrocytes in response to HIV-1 and viral proteins can cause excess extracellular glutamate, which can induce increased intracellular calcium levels in astrocytes. This alteration, in turn, can elevate even more release of glutamate from cells in an autocrine manner [64].

There is now steady accumulating evidence that HIV-1 associated excitotoxicity is a direct result of brain glutamate dysregulation. This detrimental process is believed to involve activating N-methyl-D-aspartic acid receptors (NMDARs) located in neuronal membrane by excess extracellular glutamate. The overactivation of NMDARs can cause an increase in intracellular Ca^2+^ [12] and the formation of free radical (nitro oxide and superoxide anion) [65]. The NMDAR over activation also causes activation of stress-associated protein kinases and caspases along with the production of proinflammatory lipids [66,67] and release of additional neurotransmitter glutamate resulting in further injury [67]. All of these alterations may act synergistically to contribute to HIV-1 associated neuronal injury and ultimately HAND pathogenesis.

Since glutamate excitotoxicity is a factor in HIV neurotoxicity that culminates to HAND, potential ways to regulate the activation of the glutamate system for the treatment of HAND may be beneficial for the symptoms of HAND. Many strategies have been used over the years to facilitate the treatments by inhibition of glutaminase and glutaminate carboxypeptidase (the enzymes responsible for the production of glutamate), antagonists of glutamate receptors (NMDARs, AMPA, kainate and mGluR - responsible for glutamate excitotoxicity) or activation of glutamate transporters (e.g. EAAT1 and EAAT2 - responsible for mobilizing glutamate away from the synaptic cleft) [64,68]. In addition, there is preliminary experimental evidence that these approaches have therapeutic potential for ameliorating the HAND symptom repertoire [64]. Therefore, identification of specific inhibitors and further mechanistic insights are of significant important for the development of therapeutic strategies.

## Intracellular Ca^2+^ Concentration

Ca^2+^ is a vital second messenger in astrocytes and involved in astrocyte response and communication. Ca^2+^ waves in astrocytes can transfer signals to neurons and modulate astrocyte activity [69]. Changes in the intracellular Ca^2+^ concentration play a critical role in many processes involved in the modulation of signal transduction, development and plasticity in the CNS. An increase of intracellular Ca^2+^ concentration is associated with cell excitability [70,71]. In addition, Ca^2+^ signals control many functions of astrocytes, especially exocytotic release of neurotransmitters glutamate or D-serine from astrocytes [72]. HIV-1 Tat can modulate intracellular Ca^2+^ concentration through activation of the endoplasmic reticulum (ER) pathway in astrocytic culture [73]. As a consequence, elevation of intracellular [Ca^2+^] in astrocytes is able to initiate a similar response in nearby astrocytes in a regenerative wavelike fashion. Importantly, recent data shows that astrocyte [Ca^2+^] modulates neuronal activity [74,75], and Ca^2+^ elevation in astrocytes can lead to increases in neuronal [Ca^2+^]_i_ [76]. Moreover, sustained elevation of the neuronal [Ca^2+^] may trigger excessive NMDAR activation and consequent excitotoxicity [67].

The key pathway, that leads to [Ca^2+^]_I_ change, is the involvement of glutamate receptors. It has been demonstrated that the activation of glutamate receptors such as NMDA, AMPA, kainate receptors and metabotropic glutamate receptor subclass (mGluR1 and mGluR5) can change [Ca^2+^]_i_ [77]. Released glutamate, in return, activates an inositol trisphosphate (IP_3_) pathway, which can cause calcium release from intracellular storage. This increase can then be transferred and spread to neighboring astrocytes via gap junctions [42].

Since Ca^2+^ has many unique properties such as flexible co-ordination chemistry, high affinity for carboxylate oxygen and rapid binding kinetics, Ca^2+^ is considered as one of the most popular intracellular second messengers [33]. In particular, Ca^2+^ signals control several functions of astrocytes. It takes an active part in inducing exocytotic release of neurotransmitter from astrocytes as well as contributing in HIV neurotoxicity.

## Astrocytes and Blood Brain Barrier

The blood brain barrier (BBB) is a specialized system of brain microvascular endothelial cells (BMECs) that function to separate circulating blood from brain extracellular fluid, which prevents toxic substances present in the blood from entering the brain and supplies brain tissues with nutrients [22]. The BMECs are surrounded by astrocytes and their end feet processes, which contribute importantly to the development and function of the BBB [34]. Furthermore, astrocytes are supposed to be involved in maintaining the microenvironment that preserves the functionality of BMECs [78]. It has been accepted that HIV-1 neuroinvasion occurs as a result of HIV-1-infected monocytes across the BBB, and the dysregulation of the BBB permeability during and after neuroinvasion has a critical impact on HIV-1 neuropathogenesis [22]. Therefore, the role of astrocytes in the BBB is one of the many motives to understand the molecular mechanisms associated with HIV-1 neuroinvastion and neuropathological process.

Astrocytic end feet, which contact the BBB, are rich in glutamate transporters and potassium channels. These proteins are involved in ion and glutamate homeostasis [79]. As indicated earlier, HIV-1 proteins alter glutamate uptake and potassium channel activity in astrocytes, thus, it is conceivable that this alteration may affect the BBB’s integrity. In another aspect, HIV-1 Tat and gp120 has been shown to elevate intracellular Ca^2+^. This increase in astrocytic Ca^2+^ may induce the release of COX-1 metabolic products, resulting in vasodilatation of capillaries [80]. It is reported that astrocytes secret many molecular mediators such as NO, prostaglandin (PGE), and arachidonic acid (AA) that can increase or decrease CNS blood vessel diameter and blood flow. Astrocytes also produce and release several angiogenic factors such as angiopoetin 1 and neurotrophins that potentially play a part in the development of new brain capillary functions. Notably, it has been observed that HIV-1 Tat and gp120 alter the expression of tight junction proteins (TJ) which is associated with astrocytes along the barrier, resulting in increasing permeability of the BBB [22,81]. This evidence supports the idea that astrocytes would likely contribute to the effect of HIV-1 and its proteins on the BBB integrity, resulting in increased penetration of HIV into the CNS. The possible mechanism of this contribution to HIV-1 disease progression is an important point for future investigation.

## Astrocyte-neuron Interaction

Communication between astrocytes and neurons is bidirectional. It is now accepted that astrocytes and neurons establish a highly dynamic reciprocal relationship which influences CNS growth, morphology, repair and aging. Therefore, it is highly conceivable that dysfunction of astrocytes could potentially contribute to neurological diseases. Recent studies have suggested that reactive astrocytes gain neurotoxic properties. Notably, excitotoxicity in the brain is a consequence of dysregulated mechanisms in astrocytes, which affect neurons when neither the astrocytes nor the neurons are direct targets of viral infection.

Consistent with such a variety of crucial functions exerted by astrocytes to support neurons, astrocyte impairment has been proven to contribute to neuronal dysfunction in various neurodegeneration diseases, such as amyotrophic lateral sclerosis, Huntington disease and Alzheimer’s disease [3]. *In vitro* studies have shown that Tat released from astrocytes produces mitochondrial dysfunction and cell death in neurons [82]. Infection of astrocytes by HIV-1 also leads to hijacking of protein synthesis and consequent reduction of trophic factors production [3]. In support of these findings, it has been shown that supernatant from Tat-expressing astrocytes were neurotoxic to neurons [24].

In addition to having direct impacts on synaptic transmission through release of transmitters, called gliotransmitters, astrocytes have the potential to exert long-term and powerful influences on synaptic function via release of growth factors and other bioactive molecules such as cytokines. It has been reported that HIV-1 Tat and HIV-1 gp120 activate astrocytes to secrete proimflammatory cytokines TNF-α [12], IL-1, IL-1β [83], proimflamatory chemokines MCP-1 and IP-10, as well as neurotoxic nitro oxide (NO) [2]. Cytokines, such as TNF-α, have been found to influence homeostatic synaptic scaling by inducing the addition of AMPA receptors, an ionotropic transmembrane receptor for glutamate, at post-synaptic membranes. Moreover, reactive astrocytes hinder regeneration of damaged neuronal circuits by secreting neuron-developmental inhibitor and glycosaminoglycans, which physically block growth cone extension [84]. All of which can potentially contribute to the inflammatory brain injury [85].

## Summary and Prospects

**Figure 1:**
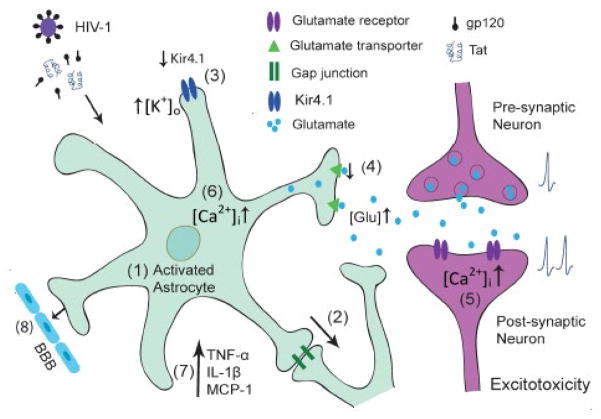
Schematic representation of astrocytic dysfunctions in HIV-1 neurotoxicity. (1) Astrocytes respond to HIV infection and viral proteins by rapid activation. (2) This response spreads and amplifies to surrounding astrocytes via gap junctions, which take part in modulating ATP release and glutamate and potassium homeostasis. (3) Reduced Kir4.1 expression and function impairs the ability of activated astrocytes to remove K^+^ in extracellular space and cause an increase in [K^+^]_0_, which would induce the depolarization of neuronal membranes and the interruption of axonal conduction and synaptic transmission. (4) Down regulation of glutamate expression and function results in glutamate excitotoxicity, which eventually may lead to neuronal injury and death. (5) This detrimental process is induced by overactivation of glutamate receptors located in neuronal membranes and causes an increase in intracellular Ca^2+^ levels, resulting in further injury. (6) Elevation of intracellular Ca^2+^ in astrocytes can also lead to increase in neuronal Ca^2+^, which may trigger excessive glutamate receptor activation and consequent excitotoxicity. (7) In addition, HIV-1 induced activated astrocytes secrete proimflammatory cytokines and chemokines, which may cause neuronal dysfunction or injury. (8) Reduced glutamate uptake, impaired potassium channel activity and elevated intracellular Ca^2+^ affect the BBB integrity and permeability resulting in increased penetration of HIV into the CNS. These alterations are likely acting synergistically to contribute to the accumulation of neurotoxicity and potentially play a role in HIV-1 induced neuropathogenesis.

In the brain, astrocytes represent a main population of the non-neuronal cell type and fulfill a crucial role in both physiological and pathological conditions. In a physiological environment, astrocytes regulate brain homeostasis, synaptic transmission and plasticity to protect neurons against toxic compounds, and support metabolically to ensure their proper functioning. Under pathological condition such as HAND, astrocytes are involved in HIV-1 neuropathogenesis. A series of changes in astrocyte functions are detected during HIV-1 infection including, but not limited to, astrocytosis, reduced glutamate uptake and altered ion homeostasis (Figure 1), which may be amplified via gap junctions to surrounding astrocytes. As astrocytes are in close contact with neurons and are capable of sensing neuronal activity, the impairment on aforementioned astrocyte functions can cause neuronal dysfunction or injury by increased extracellular K^+^ and glutamate concentration or intracellular Ca^2+^ concentration of neurons, or by release of proinflammatory chemokines (Figure 1), eventually development of HAND. Therefore, modulation of astrocyte functions may have therapeutic potential for HIV-1-associated neurological disorders as well as other neurodegenerative disorders in which astrocyte dysfunction plays a role in the pathogenesis.
